# Children’s Hospital of Philadelphia

**Published:** 1860-07

**Authors:** 


CHILDREN'S HOSPITAL OF PHILADELPHIA.—The Annual Report of this Institution shows that the number of children under treatment during the last year was 5019, 124 having been in-door cases. What a field for the study of infantile pathology and practice! The attending physicians of the hospital are, T. H. Bache, Francis W. Lewis, R. A. F. Penrose, and W. R. Dunton; the consulting physicians, William Pepper and J. Forsyth Meigs; and the consulting surgeon, George W. Norris.

